# Mechanical unfolding reveals stable 3-helix intermediates in talin and α-catenin

**DOI:** 10.1371/journal.pcbi.1006126

**Published:** 2018-04-26

**Authors:** Vasyl V. Mykuliak, Alexander William M. Haining, Magdaléna von Essen, Armando del Río Hernández, Vesa P. Hytönen

**Affiliations:** 1 Faculty of Medicine and Life Sciences and BioMediTech, University of Tampere, Finland and Fimlab Laboratories, Tampere, Finland; 2 Cellular and Molecular Biomechanics Laboratory, Department of Bioengineering, Imperial College London, London, United Kingdom; University of Uppsala, SWEDEN

## Abstract

Mechanical stability is a key feature in the regulation of structural scaffolding proteins and their functions. Despite the abundance of α-helical structures among the human proteome and their undisputed importance in health and disease, the fundamental principles of their behavior under mechanical load are poorly understood. Talin and α-catenin are two key molecules in focal adhesions and adherens junctions, respectively. In this study, we used a combination of atomistic steered molecular dynamics (SMD) simulations, polyprotein engineering, and single-molecule atomic force microscopy (smAFM) to investigate unfolding of these proteins. SMD simulations revealed that talin rod α-helix bundles as well as α-catenin α-helix domains unfold through stable 3-helix intermediates. While the 5-helix bundles were found to be mechanically stable, a second stable conformation corresponding to the 3-helix state was revealed. Mechanically weaker 4-helix bundles easily unfolded into a stable 3-helix conformation. The results of smAFM experiments were in agreement with the findings of the computational simulations. The disulfide clamp mutants, designed to protect the stable state, support the 3-helix intermediate model in both experimental and computational setups. As a result, multiple discrete unfolding intermediate states in the talin and α-catenin unfolding pathway were discovered. Better understanding of the mechanical unfolding mechanism of α-helix proteins is a key step towards comprehensive models describing the mechanoregulation of proteins.

## Author summary

In order to migrate and survive, most cells need to be attached to their environment. Cells anchor to the extracellular matrix via transmembrane integrin, connecting it to contractile the cytoskeleton. Similarly, cell-cell contacts are formed via transmembrane cadherin, which also connects to the contractile cytoskeleton through scaffolding proteins. Examples of such proteins include talin and α-catenin, which connect integrin and cadherin respectively, to actin filaments of the cytoskeleton. Mechanical forces that are transmitted between the cell and its environment activate binding and regulate the functions of these scaffolding proteins at cell-extracellular matrix and cell-cell contacts. Functions of talin and α-catenin are tightly modulated by mechanical forces. The stretching of these proteins under mechanical load exposes buried binding sites for other partners, such as vinculin. We used steered molecular dynamics simulations and single-molecule atomic force microscopy to study how these proteins unfold under load. Our results suggest that α-helical talin and α-catenin unfold through stable 3-helix intermediates. These intermediates represent biologically active states, which may allow recruitment of other binding partners.

## Introduction

Protein activity can be modulated by mechanical cues in addition to chemical stimuli and ligand binding. Mechanical forces can induce conformational changes in the protein structure, that leads to either a switch between the functional states of the protein, or allows for multiple functions[1,2].

α-helix folds are highly abundant among the structural proteins located at the cell-cell and cell-ECM contacts [3–5], among the essential muscle costamere complexes[6], as well as among the structures interconnecting the cytoskeleton[7,8] and cellular organelles[9]. Despite the numerous studies on mechanotransduction and the mechanosensitivity of proteins, the mechanisms associated with forced protein unfolding and mechanosignaling are not well understood especially where α-helical proteins are concerned. One reason for such a lack of understanding might be that α-helices are, in general, mechanically weaker compared to β-strand folds[10] and are therefore challenging to study experimentally. The low mechanical stability of α-helical proteins requires sophisticated experimental methods capable of force measurement in the range of piconewtons[11].

Among the α-helical structural proteins, several distinct folds have been identified. Talin-like proteins contain 5- and 4-helix bundles[12]. Proteins of the catenin family contain a 4-helix conformation with long α-helices interconnecting two bundles[13]. Both of these multi-helical protein folds respond to mechanical load by a dissociation of the bundles leading to mechanoregulatory function. The spectrin fold is formed of a 3-helix conformation with a long α-helix connecting the neighboring domains, forming a rigid rod of interacting 3-helix bundles. The spectrin-like conformation accounts for structural reinforcement in the cellular scaffolds[3,4,7]. Finally, single long α-helices and coils can also be found among structural α-helical proteins (PDB id 5KHT).

Focal adhesions and adherens junctions are fundamental mechanosensitive structures through which cells communicate with the extracellular matrix and with their adjacent cells, respectively. The processes of focal adhesion formation and maturation are regulated by mechanical signals[14,15]. Similar to the role of focal adhesions in the cell-ECM connection, adherens junctions are essential for cell-cell contacts. Adherens junctions are associated with the cadherin super-family of transmembrane proteins, which are connected through catenin-rich protein complexes to the actin cytoskeleton. The cadherins bind to the cytoplasmic protein β-catenin, which in turn binds to the filamentous F-actin binding adaptor protein, α-catenin[16,17].

**Fig 1:**
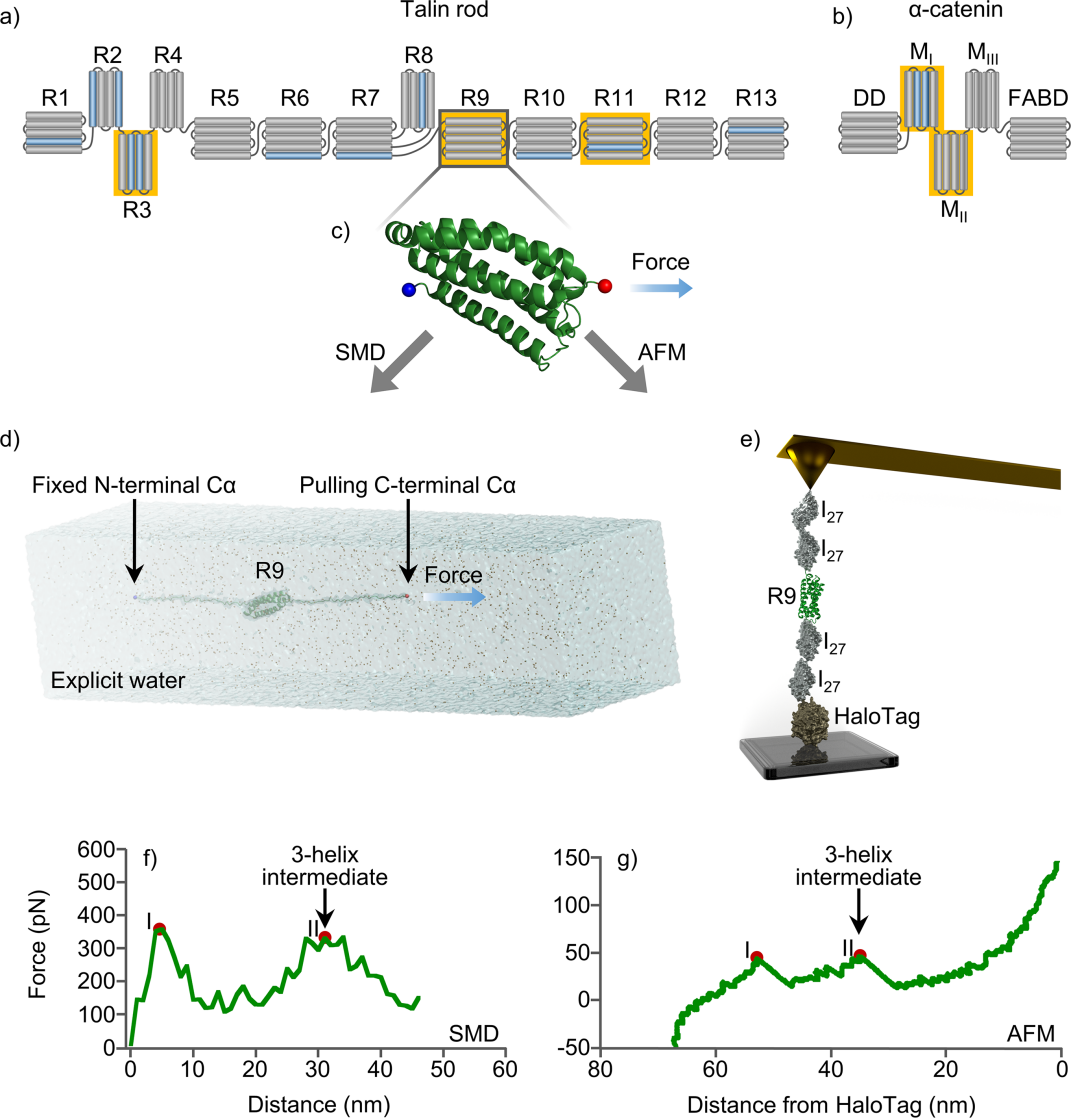
α-helix bundle mechanical stability. (a) Schematic representation of talin rod bundles and (b) α-catenin domains. Vinculin binding sites colored in blue. (c) Cartoon illustration of R9, where Cα of N-terminal residue colored in blue and Cα of C-terminal residue is red. (d) SMD water box and (e) smAFMsetup, that were used for end-to-end stretching of studied proteins. Unfolding force profiles of talin rod R9 in (f) SMD and (g) smAFM experiments, where after collapsing of 5-helix bundle (peak I) the stable 3-helix intermediate was found (peak II). α-helix bundles used in unfolding experiments highlighted with yellow.

Talin is a large focal adhesion protein that contains an N-terminal head domain, which is responsible for integrin binding. The larger talin rod domain consists of amphipathic α-helices arranged into 13 four or five-helix bundles (R1–R13) and a single helix dimerization domain (DD) at the C-terminal end (Fig 1A). Talin provides a link between the ECM, via the talin head-integrin interaction, and the cytoskeleton through the binding of actin filaments at actin binding sites located in the rod domain. Furthermore, the talin rod comprises up to 11 buried vinculin binding sites (VBSs), distributed along its structure. These binding sites are exposed by partial or complete unfolding of different bundles[18,19] as a result of mechanical stretching. In this way mechanical load regulates talin function by exposing the buried binding sites for certain binding partners such as vinculin[20], while simultaneously, epitopes for other binding partners become inactivated. An example of such epitope inactivation would be the talin binding partner RIAM which binds only to folded talin domains[21]. Thus, conformational changes of talin under mechanical load regulate the recruitment and activation of talin-interacting proteins. Interestingly, talin dimerization is also regulated by mechanical force [22].This property highlights talin as a key player in the transmission and sensing of mechanical signals between the extracellular matrix and cell cytoskeleton. These mechanical signals are central for a variety of cellular functions including spreading, migration, invasion and substrate sensing[23–25].

Similar to talin, α-catenin recruits vinculin, providing the mechanical connection between cell-cell adhesions and the cytoskeleton. α-catenin contains 5 α-helix domains: the dimerization domain (DD) functioning as β-catenin binding domain, three modulation domains I, II and III (M_I_, M_II_ and M_III_), and an F-actin binding domain (FABD) (Fig 1B). Vinculin binds the M_I_ domain of α-catenin while the two adjacent domains (M_II_ and M_III_) inhibit vinculin binding to M_I_. It has been demonstrated that vinculin is recruited in a force-dependent manner to the cadherin/catenin complex upon force-dependent unfolding of α-catenin. This process resembles the binding of vinculin to the talin rod domain upon tension-dependent unfolding. This tension-dependent unfolding is thought to be central in cell-cell adhesion mechanosensing[9,26,27].

It has been previously hypothesized that the force-dependent unfolding of the vinculin binding domains of the talin rod and the α-catenin domains includes stable intermediate conformations[28–30]. However, this has never been previously studied in detail in computational simulation or in experimental setup suitable for analysis of single-molecule unfolding events. α-helical proteins are associated with key physiological and pathological processes in mechanobiology[31–33]. A number of diverse diseases from heart[34] and muscle[35], diseases of bone, vascular and nervous system, or skin[36] have been associated with mechanobiology. Hence, better understanding of their unfolding characteristics will open the possibility of answering a plethora of questions in biology and medicine. In this study, we investigate the molecular mechanisms of the unfolding of two α-helical proteins, talin and α-catenin, on an atomistic level by a combination of steered molecular dynamics (SMD) simulations and single-molecule atomic force microscopy (smAFM).

This study elaborates in closer detail on observations made during our previous work described in Haining et al. [28]. Previously, we have concentrated on the mechanical sensitivity of the talin rod subdomains during the initial domain breaking. We have reported that the talin subdomains are similar, however not identical in their ability to withstand mechanical load. All the tested talin subdomains unfolded in AFM setup over a range of mechanical forces between 10 and 40 pN. During the SMD simulations we repeatedly observed a possible unfolding intermediate in the unfolding trajectories which we intended to investigate further. Therefore, in the light of our previous findings, we have now selected two talin subdomains on either end of the mechanical sensitivity scale for the current study; weak R3 and strong R9. Talin R9 is an exceptional subdomain responsible for talin autoinhibition while it does not contain VBS. For that reason, we have also included subdomain R11 to investigate the unfolding intermediate in the terms of VBS activation.

## Results

In order to probe the existence of stable intermediates in the unfolding trajectory of α-helical mechanosensitive domains, we have selected two mechanoregulatory proteins, talin and α-catenin (Fig 1). We have selected the mechanically stronger talin 5-helix bundles R9 and R11, and the mechanically weaker 4-helix bundles of talin R3 and α-catenin modulation domains I to II (M_I_-M_II_).

### Constant velocity pulling simulations reveal that talin rod bundles unfold through stable 3-helix intermediates

**Fig 2:**
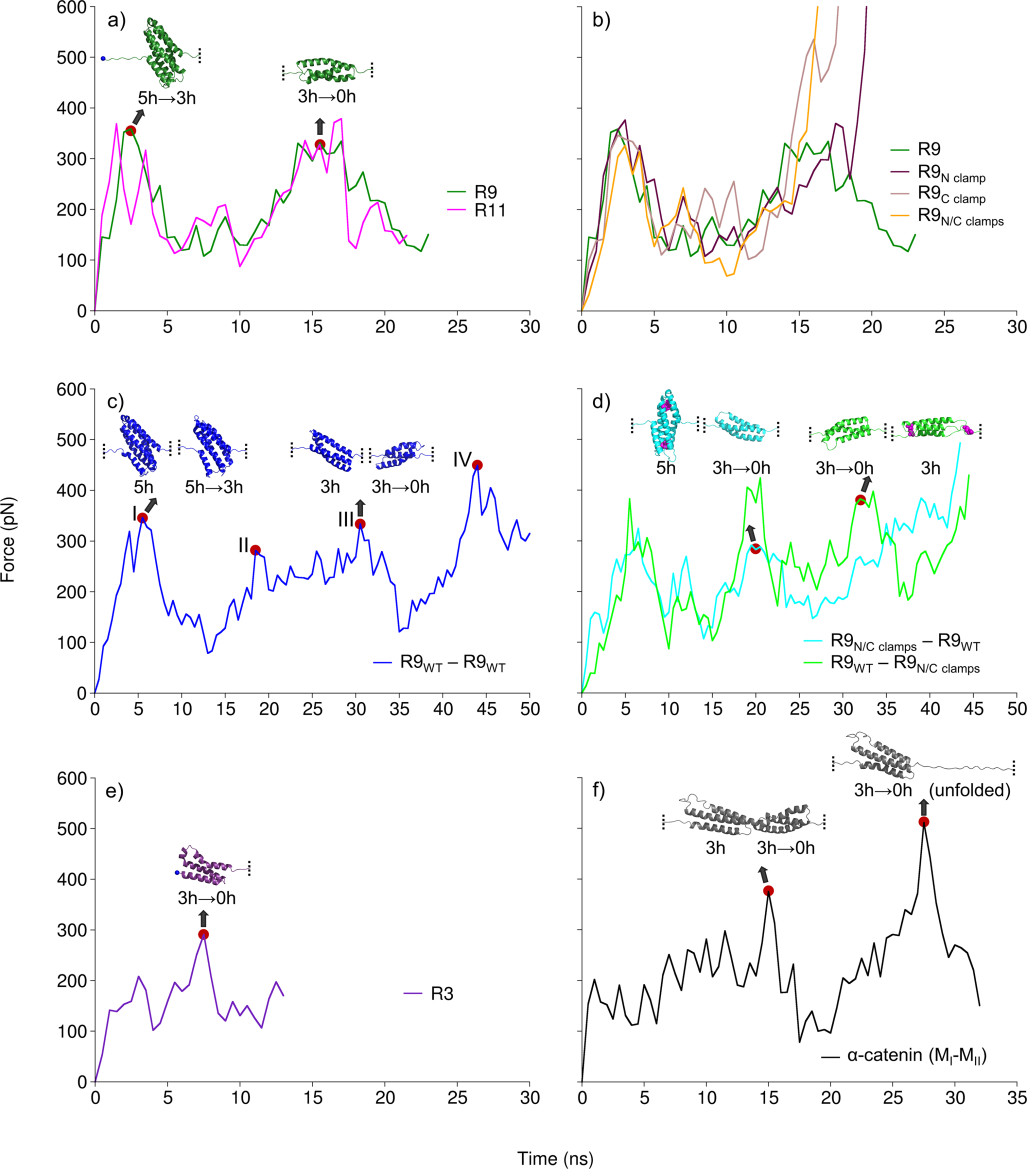
Unfolding force profiles of the studied protein constructs in constant velocity SMD. (a) Unfolding forces for 5-helix talin rod R9 and R11 have similar profiles and show two peaks, which correspond to breaking of 5-helix and 3-helix state respectively. (b) R9 constructs with disulphide clamps have similar force profiles to wild-type R9, but unfolding of 3-helix state was blocked. Tandem constructs for (c & d) R9 and (f) α-catenin (M_I_-M_II_) were unfolded through 3-helix state for both monomers simultaneously. (c) R9 (wt)–R9 (wt) tandem showed four peaks, corresponding to breaking of the 5h & 5h→3h (peak I), 5h→3h & 3h (peak II), 3h & 3h→0h (peak III), and 3h→0h & 0h (peak IV) respectively. (d) Tandems with the clamped 3-helix state in one monomer showed three peaks, lacking the peak for unfolding of the clamped 3-helix state. (e) Unfolding force for 4-helix R3 bundle has one peak that corresponds to collapsing of 3-helix state. (f) 4-helix α-catenin showed one peak for breaking the 3-helix state in each domain. Cysteine residues in (d) R9 tandems, that form disulphide clamps shown as magenta spheres. Structure snapshots correspond to force peaks highlighted with red dots.

**Fig 3:**
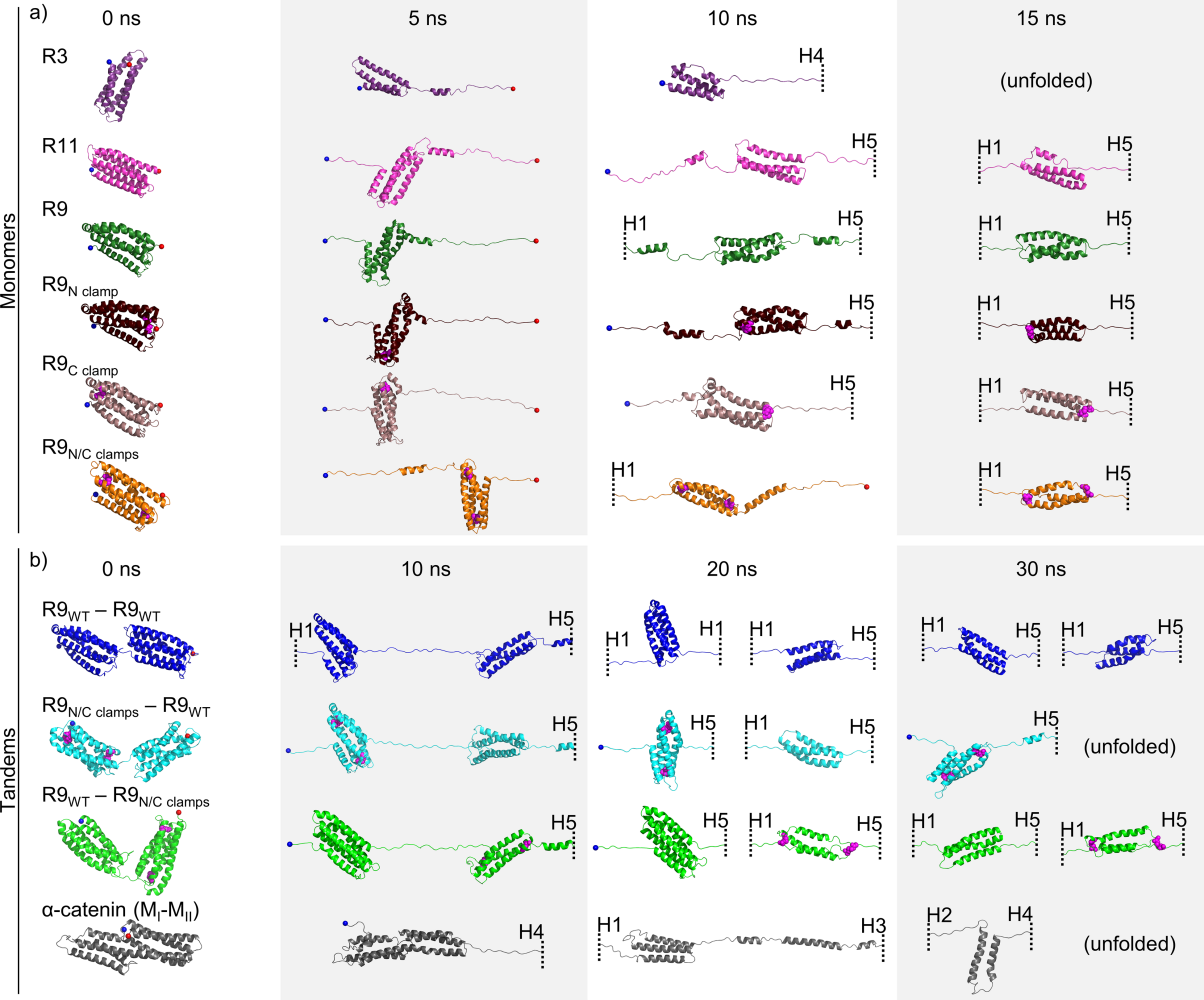
Representative structure snapshots from SMD. Talin rod domain bundles and α-catenin (M_I_-M_II_) in constant velocity SMD simulations. Structures were taken (a) at 0, 5, 10 and 15 ns for monomers, and (b) at 0, 10, 20 and 30 ns for tandem constructs. Cysteine residues forming the disulphide clamps shown as magenta spheres. Unfolded structures are cut away and presented by dashed line with a helix identifier.

We subjected two talin rod 5-helix bundles, R9 and R11, to end-to-end SMD stretching with constant velocity pulling simulation at 2 nm/ns. Previously, talin 5-helix bundles were more mechanically stable in our SMD simulations compared to 4-helix bundles. According to our previous study[28], R9 was one of the strongest rod bundles. Overall, unfolding of R9 and R11 showed two force peaks (Fig 2A), which correspond to breaking of the 5-helix bundle and 3-helix intermediate. This unfolding intermediate consists of core helices (H2–H4) after the dissociation of H1 and H5. For R9 the maximum unfolding forces detected in constant velocity pulling simulation at 2 nm/ns were 348 ± 24 pN and 349 ±39 pN (average force ± standard deviation). Unfolding peak forces for R11 were very similar to those for R9 (Fig 2A). Representative snapshots of the unfolding trajectories are shown in Fig 3, indicating changing of the bundle conformation during unfolding.

To confirm the unfolding intermediate, we designed point mutations in the R9 bundle forming disulfide bonds (clamps) in order to block the unfolding of the 3-helix core. These clamped R9 mutants were subjected to end-to-end pulling in simulation and experimental setup. We prepared three constructs including L1698C and A1748C cysteine mutations that protect 3-helix core from N-terminus (N clamp), A1720C and A1779C mutations that prevent unfolding of the 3-helix core from C-terminus (C clamp), and R9 construct with both N- and C-terminal disulfide clamps (N/C clamps) (S1 Fig). All R9 clamp mutants showed very similar unfolding of 5-helix state compared to the wild-type R9 (Fig 2B). The unfolding of the 3-helix state was effectively blocked in the R9 equipped with N/C clamps, while the constructs with only one disulfide clamp allowed unfolding of either H2 (R9 with C clamp) or H4 (R9 with N clamp) of the 3-helix core, but did not compromise the stability of the 3-helix intermediate (Fig 2B).

In additional experiments, we investigated talin R3 bundle and α-catenin modulation domains I to II (M_I_-M_II_), which are all 4-helix bundles. Talin R3 was easily unfolded to the 3-helix state by the separation of H4. The 3-helix intermediate (H1–H3) was more stable compared to the 4-helix bundle, the unfolding peak force for breaking the R3 3-helix state was 276 ± 20 pN (Fig 2E). Similarly, α-catenin was unfolded to 3-helix conformation by dissociation of H4 (four out of five simulations) in M_II_ and H1 in M_I_ (all five simulations). Further unfolding showed two force peaks for breaking the 3-helix state in M_II_ (at 349 ± 33 pN) and M_I_ (at 461 ± 68 pN) (Fig 2F). Collectively these data suggest that both 4- and 5-helix bundles unfold through stable 3-helix intermediate state. Furthermore, 5-helix bundles withstand mechanical load better than 4-helix domains, which are easily unfolded to the 3-helix state.

Described constant velocity SMD simulations of individual talin rod bundles and α-catenin were run five times each. Unfolding force profiles showed that our results are well reproducible (S2 Fig) and allowed us to calculate average peak force and a standard deviation (S1 Table).

### Talin tandem domain experiments confirm the existence of 3-helix intermediates

To assess the effects of force penetration on the unfolding mechanisms in SMD simulations, we studied the unfolding mechanisms and the existence of the stable intermediates in linear protein chain consisting of two talin R9 monomers resembling the natural biological assembly of talin. We designed tandem construct possessing exactly the same mechanical stability, i.e. two talin R9 domains (R9_WT_−R9_WT_ tandem). Furthermore, we analyzed the R9 tandems with disulfide clamps, protecting the 3-helix core from unfolding, in either first or second monomer, with respect to the fixed N-terminal and pulled C-terminal end. Thus, we prepared two tandem constructs with clamps, R9_N/C clamps_−R9_WT_ and R9_WT_−R9_N/C clamps_, respectively. For R9_WT_−R9_WT_ tandem, unfolding force showed four peaks, corresponding to the breaking of 5-helix states first, followed by dissociation of the 3-helix states in both α-helix bundles (Fig 2C). Because the pulling was applied to Cα atom of C-terminal residue, the second R9 in the tandem was closest to the point of pulling and unfolded to 3-helix state first. Both monomers of the R9_WT_−R9_WT_ tandem unfolded to the 3-helix intermediate within approx. 30 ns of the SMD simulation with 2 nm/ns pulling velocity, and unfolded at approx. 50 ns. Both tandems containing one clamped monomer showed three unfolding force peaks (Fig 2D) lacking the peak corresponding to the breaking of the disulfide-clamped 3-helix structure. Indeed, the force penetration did affect the two different tandems with clamps resulting in different unfolding trajectories. For R9_WT_−R9_N/C clamps_ tandem, both monomers had 3-helix conformations at ~ 30 ns, while for R9_N/C clamps_−R9_WT_, closest to the point of pulling monomer (R9_WT_) unfolded completely before R9_N/C clamps_ molecule unfolded to 3-helix state (Fig 2D, Fig 3).

The investigation of the unfolding of a tandem provided us with a tool of studying the unfolding principles of multiple domains. Furthermore, the use of disulfide clamps in the tandem construct protecting the stable state provided us with a comparison force trace and additional proof of an intermediate unfolding conformation in both mechanosensitive proteins. Altogether, these findings indicate that the unfolding force required for the unfolding of the 3-helix intermediate state is similar to that needed for the unfolding of 5-helix state.

### smAFM analysis of α-helical bundles confirms unfolding through intermediate states consistent with 3-helix pattern shown by SMD

**Fig 4:**
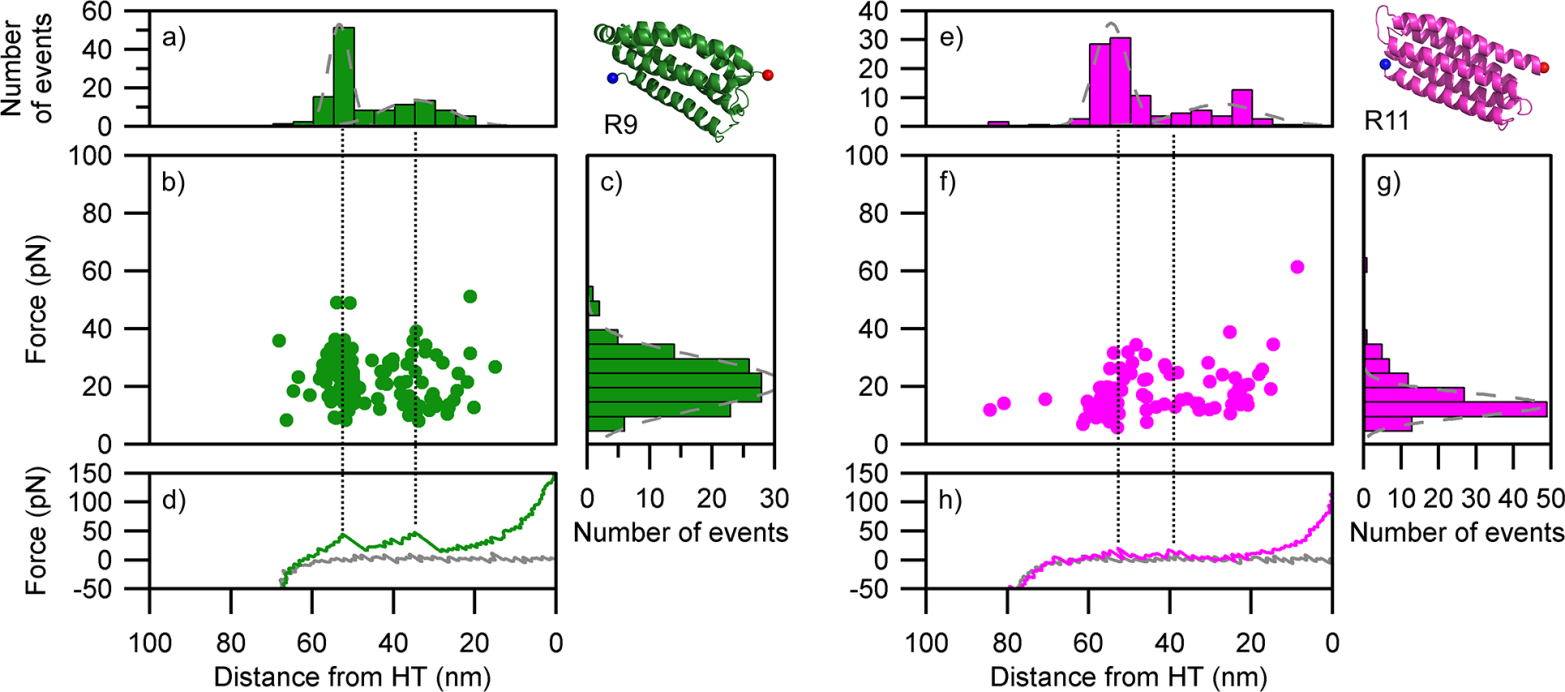
Unfolding patterns of the α-helix monomers as determined by smAFM. (a-d) R9 (87 traces). (e-h) R11 (79 traces). (a & e) length histogram indicating the distance from unfolding event to HaloTag standard; (b & f) scatter plot showing force vs position for all unfolding events; (c & g) histogram the force associated with unfolding events; (d & h) representative force extension retraction curve (green & magenta line) showing how the unfolding events relate to scatter plot and length histogram (black dotted line), gray line represents approach curve for AFM tip. Gray dotted lines on (c & g) histograms represent Gaussian fits.

We utilized smAFM to characterize the unfolding patterns of R9 and R11 constructs and captured the 3-helix intermediate (Fig 4). Similarly to the SMD, we detected two unfolding events for each of the bundles, implying that unfolding occurs through a mechanically stable intermediate. Overall the bundle stability was higher in the case of R9 than R11, consistent with our previous results[28]. The distance between the two unfolding events is 20–25 nm, which is consistent with the collapse from 5 helices to 3 helices. Likewise, the distance from the second unfolding event to the HaloTag ruler of 25–35 nm is consistent with the subsequent collapse of the 3-helix intermediate. As such the smAFM data supports the picture derived from the SMD analysis: 5-helix bundles collapse via a stable 3-helix intermediate.

**Fig 5:**
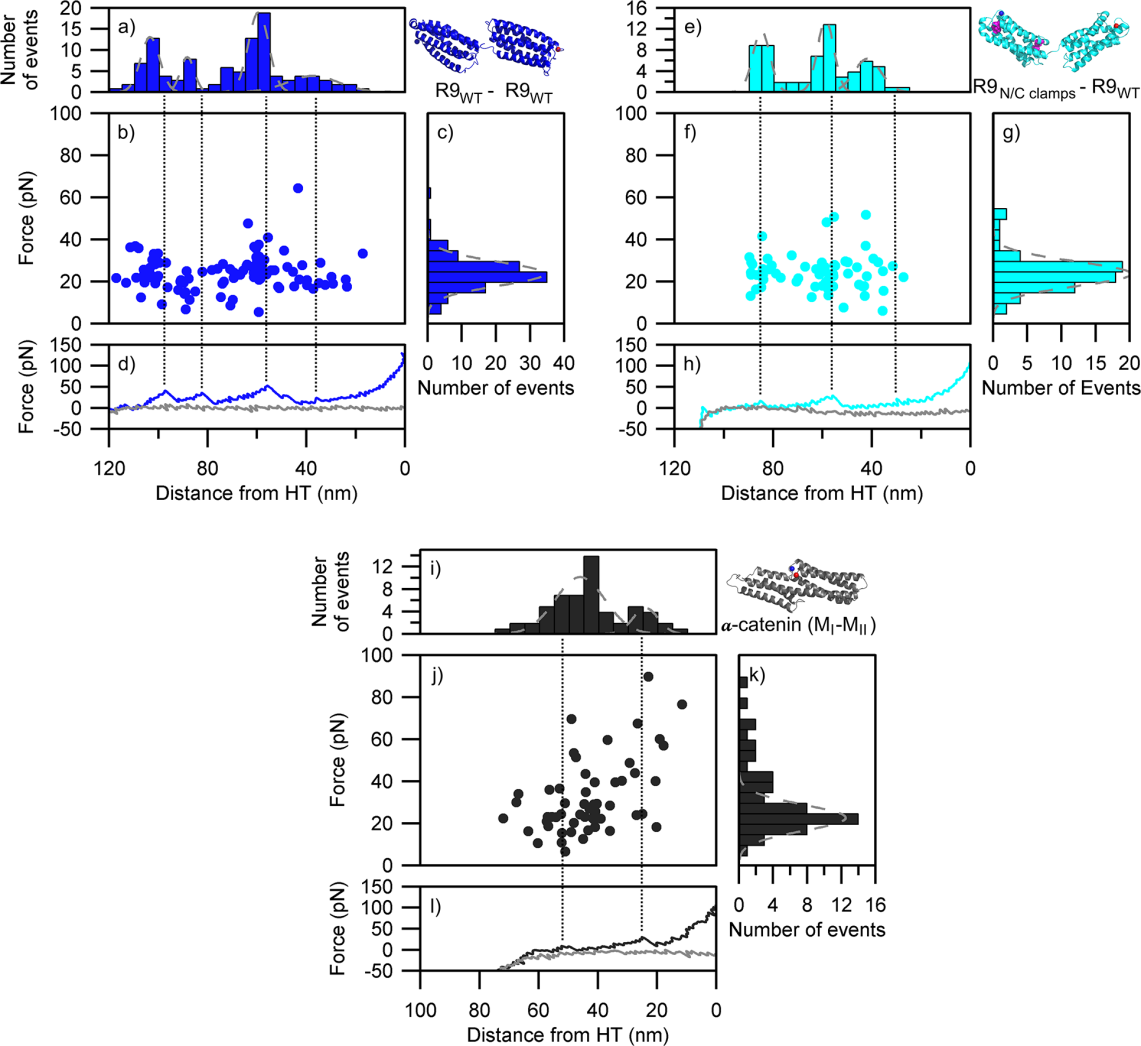
Unfolding patterns of tandem α-helical bundles as determined by smAFM. (a-d) Talin R9 (wt)–R9 (wt) (47 traces). (e-h) R9 (N/C-clamps)–R9 (wt) (34 traces). (i-l) α-catenin (M_I_-M_II_) (40 traces). (a, e & i) length histogram indicating the distance from unfolding event to HaloTag standard; (b, f & j) scatter plot showing force vs position for all unfolding events; histogram the force associated with unfolding events; (d,h & l) representative force extension retraction curve (blue, cyan & black line) showing how the unfolding events relate to scatter plot and length histogram (black dotted line), gray line represents approach curve for AFM tip. Gray dotted lines on (c, g & k) histograms represent Gaussian fits.

We also tested the R9 tandem construct to examine if the 3-helix intermediate could be detectable within a model of a polyprotein (Fig 5A–5D). We detected 4 unfolding events, consistent with a pattern of two bundles unfolding via a stable intermediate. The distances between the first and second event (~20 nm), second and third (~30 nm), third and fourth (~20 nm) and fourth and HaloTag (~30 nm) imply that, contrary to the SMD results, one α-helix bundle collapsed completely through a 3-helix intermediate before the second α-helix domain started unfolding. However, it is difficult to be certain, given the error margin of the peak locations and the likely stochastic nature of the unfolding process. When the 3-helix disulfide clamp was introduced into one of the tandem R9 domains, we saw a reduction in the number of unfolding events from 4 to 3 (Fig 5E–5H). This, along with the reduction of the overall unfolding length from 105 nm to 85 nm, demonstrates that the disulfide clamping was able to protect the 3-helix intermediate of the R9 from mechanical unfolding.

The forced unfolding of α-catenin modulation domains I to II (M_I_-M_II_) by AFM produced a pattern consistent with the SMD simulation (Fig 5I–5L). We detected two unfolding events with a maximum unfolding length of ~50 nm which implies that there is no unfolding event with respect to the reduction of both 4-helix bundles into 3-helix states. This is in agreement with herein presented SMD data and with our previous work reporting on the lower mechanical stability of the 4-helix bundles[28]. The two events observed with unfolding lengths of ~25 nm correspond to the mechanical unfolding of 3-helix intermediate states.

### Constant force SMD confirms the existence of 3-helix intermediates in talin rod R3 and R9

**Fig 6:**
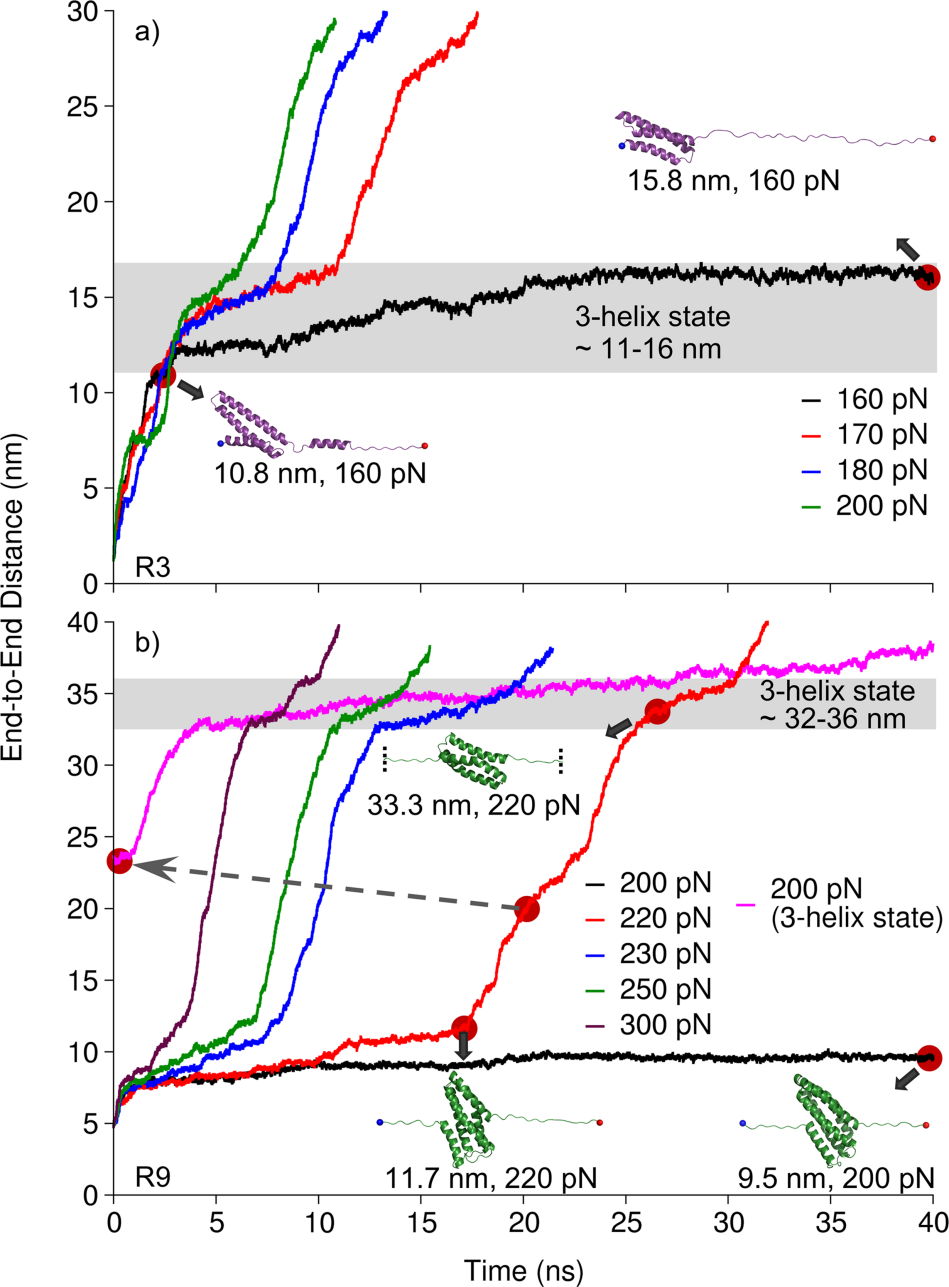
Stability of the intermediate states. Talin rod (a) R3 and (b) R9 over time in constant force SMD using constant force pulling at different force regimes. R9 is a strong 5-helix bundle, while stable 3-helix intermediates were observed in both R3 and R9 (gray shading). The pink trace in panel (b) corresponds to end-to-end distance measured under 200 pN applied force, where 220 pN simulation was used as a starting structure after passing the 5h to 3h transition (dashed gray arrow).

For the comparison of the 3-helix state mechanical stability we selected two mechanically diverse talin rod bundles, i.e. the mechanically weaker R3 and the mechanically stronger R9 for more detailed analysis. Although constant velocity SMD simulations are an excellent tool comparing the results with AFM analysis, they are less sensitive for the assessment of intermediate states as compared to constant force simulations. Therefore, we subjected the R3 and R9 bundles to constant force pulling simulations where, after screening of suitable force regime, constant force ranging from 160 pN to 200 pN for R3, and from 200 pN to 300 pN for R9 (Fig 6) was used. In constant force SMD, 4-helix R3 was weak even at 160 pN and rapidly unfolded to 3-helix state (within ~ 3 ns). After the separation of H4, H3 was slowly sliding relative to H1 and H2 (from ~ 3 ns to ~ 23 ns). The 3-helix intermediate did not unfold at 160 pN in 40 ns time window (Fig 6A), however, it unfolded completely (at ~ 72 ns) in extended 160 pN simulation (S3A Fig). R3 was extended also with constant force at 170 pN, 180 pN and 200 pN. Although it was completely unfolded after ~ 18 ns, ~ 13 ns and ~ 11 ns respectively, the stable 3-helix intermediate was observed in all trajectories (Fig 6). For strong R9 bundle, we first applied constant force of 200 pN and observed only partial uncoiling of terminal helices (H1 and H5) within 40 ns time window (Fig 6B), yet the 5-helix state remained intact. However, R9 unfolded to the 3-helix state (at ~ 86 ns) in extended 200 pN simulation (S3B Fig). The application of constant force of 220 pN or higher resulted in gradual unfolding of the bundle. During the unfolding, we recognized two stable states (5-helix and 3-helix states). In order to compare mechanical stability of 5- and 3-helix states, we used the disrupted 5-helix state of R9 and subjected it to stretching with constant force of 200 pN. Although 3-helix intermediate was relatively stable, it slowly unfolded over time under 200 pN while 5-helix bundle resisted the unfolding under the same force magnitude.

These results suggest that 3-helix state in R9 is a stable conformation. However, it is weaker as compared to the 5-helix state of R9. In more detail, the 3-helix state can be unfolded under lower force load once the 5-helix state of the R9 bundle is broken. On the other hand, the 3-helix intermediate state in R3 is the most stable conformation of the R3 bundle.

## Discussion

Numerous studies concerning α-helical mechanosensitive proteins have provided information on mechanically regulated switches between diverse binding partners and their associated functions. Perhaps one of the best known examples of this mechanoregulated protein-protein interaction is the tandem talin-vinculin, where mechanical stress applied to talin rod exposes binding sites for vinculin[37,38].

Our observation of SMD trajectories for talin multidomain constructs, described in our previous work[28], revealed a possible stable 3-helix intermediate during the forced domain unfolding. Because of the complexity of the SMD and smAFM data we obtained during the multidomain construct unfolding, we were not able to identify previously the 3-helix intermediate among the force traces directly. However, simultaneous domain unfolding was recognized for bundles of similar mechanostability in smAFM[28].

In this study, we investigated the detailed unfolding mechanisms of α-helical talin rod bundles and α-catenin M_I_-M_II_ domains to probe the presence of any intermediate or partial unfolded states. Our results show that the two studied proteins unfold through a stable 3-helix intermediate. Constant velocity pulling used in SMD and smAFM revealed, that the unfolding force profiles for the 5-helix rod bundles R9 and R11 have two peaks, which correspond to the breaking of the 5- and 3-helix states (Fig 2A). The talin 4-helix bundle R3 and α-catenin bundles M_I_-M_II_ also unfolded through stable 3-helix intermediates (Fig 2E and 2F). In addition, this 3-helix state was recognized as the most mechanically stable conformation for the 4-helix domains (Fig 6A).

Other studies have also provided indirect evidence of unfolding intermediates in alpha helical proteins. Investigations of the talin R3 subdomain have revealed a possible 3-helix intermediate capable of rapid or instant activation for vinculin binding. Specifically, the deletion of helix 4 of rod subdomain R3 (ΔR3H4) leads to super-active R3 localizing efficiently in cell-ECM contacts (S6 Fig). Rahikainen et al., 2017[23] showed that one or two destabilizing mutations in R3 H1 were sufficient to facilitate bundle unfolding, increasing the activation of vinculin binding and resulting in a strong cellular phenotype. The phenotype of the further destabilized state modified with four mutations was comparable to the super-active R3 potentially indicating a 3-helix intermediate. Further evidence of a 3-helix state in R3 domain is found in a recent study by Baxter et al. [39]. A 3-helix open state has been recognized after the dissociation of H1 from the R3 bundle under high pressure conditions. Similar effects were observed even for the talin R1 bundle, where the deletion of H5 resulted in the exposure of the VBS located in H4 and an active conformation of R1[40]. The authors also suggest that the deletion of H5, resulting in a 4-helix partial bundle, causes a destabilization of the R1 domain leading to partial unfolding. This observation is in line with our results; we showed that the 4-helix fold is a fragile conformation which does not require excessive mechanical force to unfold to a stable 3-helix state (Fig 2E). Finally, even previous computational studies suggested that only partial unfolding of talin subdomains described by minimal protein extension is sufficient for VBS activation. In more detail, R1 VBS was activated through torsional conformational change of the hydrophobic core orientation within R1 subdomain during an extension of less than 2 Å [41].

Inspection of the molecular characteristics of 5- (4- in R3) and 3-helix states did not reveal any dominant differences between these assemblies in terms of interactions or packing. The hydrophobic interactions appear to be the main factor in maintaining both these states, as shown in S4 Fig. In the previous study[28], we proposed two conserved residues that are important for maintaining R9 5-helix bundle stability, namely Leu1668 in H1 and Met1803 in H5. Further studies including experimental investigation of subdomains carrying mutations targeting the 3-helix core fragment would be needed to evaluate the contributions of individual residues for the mechanical stability of the 3-helix intermediate state.

Further unfolding of the 3-helix intermediate was observed in our experimental and simulation setup. Whether the complete unfolding of an α-helical domain takes place *in vivo*, or whether the 3-helix state is the final unfolding conformation remains unclear. Hints of both of these options can be found in the literature. As discussed earlier[40], S6 Fig, the deletion of terminal helix in R1 and R3 is sufficient for VBS activation and vinculin recruitment. Thus, we speculate that the 3-helix state is capable of vinculin binding. Vinculin binding to unfolded talin or α-catenin domains inhibits domain refolding under low mechanical load[20,27,42]. Simultaneously, vinculin binding to the 3-helix state bundle may protect it from complete unfolding[43]. Studies by Margadant et al.[43] show that the maximal length of talin is approx. 400 nm in living cells. This also supports the notion of partial unfolding even in the absence of vinculin. Interestingly, recently published work by Ringer et al.[11] revealed a force gradient across the talin rod domain. In the presence of vinculin, greater force was measured at the N-terminal end than at the C terminal end resulting in the bundle unfolding and activation for vinculin binding. As vinculin binds to activated talin and to actin, the force acting on the talin rod is divided and reduced towards the C-terminal end. We speculate that the reduced force might be insufficient to unfold the stable 5-helix subdomains located at the C-terminal end of the talin rod. However, here we showed that the intermediate resulting from 4-helix unfolding was mechanically weaker compared to the intermediate of a 5-helix bundle. Thus, the complete unfolding of 4-helix bundles at the N-terminal end of the talin rod may be possible. Based on the work by Yao et al., we may assume that complete yet reversible unfolding of R3 domain takes place under low force load. It was shown than under 4.8 pN of constant force load exerted on the full length talin, R3 occupies two distinct conformations with elongation of approx. 19 nm. However, which states these in fact are remains unclear [44]. We may hypothesize that only the end-to-end attachment to the pulling device and initial elongation under low force load is sufficient to collapse R3 into an activated 3-helix state. Such immediate conformational change would not necessarily result into an observable difference in the total end-to-end elongation compared to the R3 conformation in solution (S5 Fig) [41]. Similar elongation of approx. 19 nm between the 3-helix intermediate and the completely unfolded state was shown in our SMD results (Fig 6A). Here we also see that the 3-helix intermediate remained stable even though the end-to-end distance increased by 5 nm. This distance increase was caused by the uncoiling of H4.

**Fig 7:**
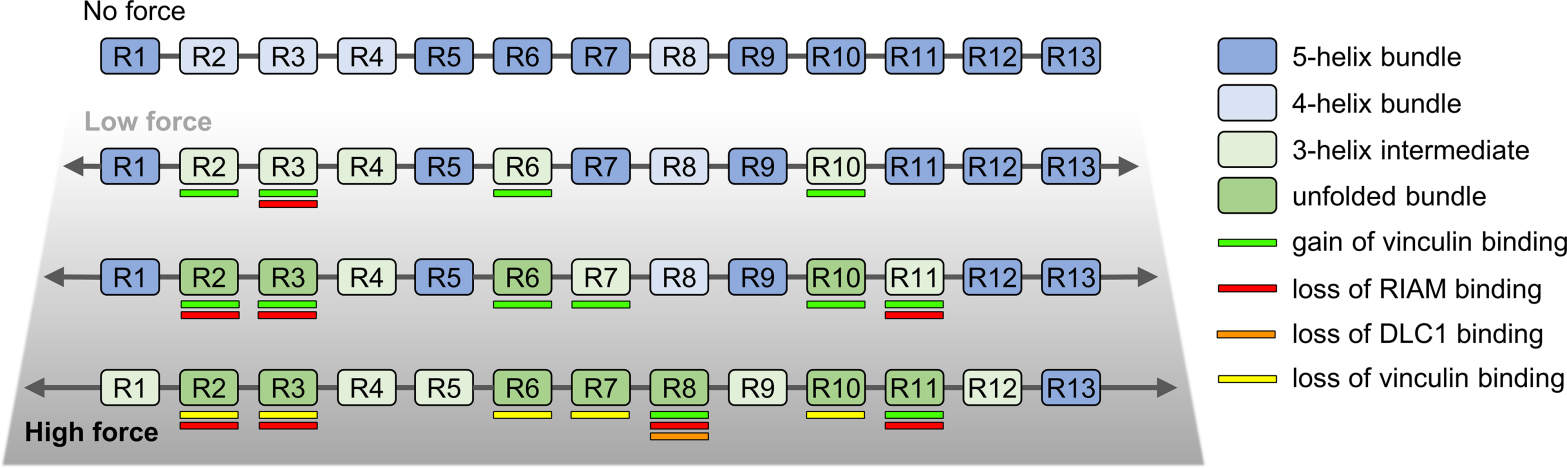
Schematic representation of talin rod unfolding through stable 3-helix intermediates. Without force, the talin rod subdomains remain intact and no VBSs are exposed. Under low load weak 4-helix bundles unfold to stable 3-helix intermediates. As the force increases, some of the 5-helix bundles unfold, forming the 3-helix intermediates along talin rod structure. Force-regulated unfolding of the talin rod changes affinity to different binding partners. RIAM and DLC1 are known to bind folded bundles, while recruitment of vinculin requires partial or complete unfolding of rod domains. VBSs that are located at terminal helices of the R2, R6, R7, and R10 bundles become available after unfolding to the 3-helix state (panel “Low force”).

Based on our observations of the talin unfolding mechanisms, we propose a model of multidomain α-helical protein unfolding under mechanical load, shown in Fig 7. In the absence of mechanical force, α-helical bundles remain in a folded conformation, capable of binding their ligands such as RIAM (R2, R3, R8 and R11) and DLC1 (R8) in the case of talin[45]. At low mechanical load, soft α-helix bundles, namely 4-helix domains, unfold to stable 3-helix intermediates. Since the activation of vinculin binding sites (VBSs) requires the unfolding of talin bundles[14], the formation of 3-helix states suggests that VBSs located at the terminal helices become available for vinculin. At the same time, the partial unfolding of the bundles leads to a disturbance of the binding sites for other binding partners located on the bundle surface, which abrogates their interaction[21]. With increasing force, additional bundles collapse to a 3-helix conformation switching from the mechanoregulatory role to a structural reinforcement role, similar to that of spectrin. In other words, it is possible that talin reacts to a range of small mechanical forces by the dissociation of certain bundles leading to a change in binding to other proteins. Such mechanoregulation would take place until the bundles reach a stable 3-helix spectrin-like conformation. At this point, the talin protein would assume a structural reinforcement role. Finally, at a high force, completely unfolded bundles would lose the ability to support ligand binding as well as structural function[20]. Such a model enables rich mechanosignaling through talin. A recent study by the Barsukov group has proposed the talin protein as being a hub for several different binding partners[45] where the proposed 3-helix intermediate state could be an essential component of binding regulation. Moreover, it has been recently shown, that the mechanical load across talin is not homogeneous, providing further variation in the regulation of talin functions[11]. Since the talin rod experiences a force gradient, once vinculin is bound, the local stress may become modulated and insufficient to unfold the 3-helix state. The 3-helix state may thus represent abundant talin rod subdomain conformation in living cells[11,43]. Experimental work is essential to confirm and refine these models.

While the activation of talin binding sites by mechanical force has been long studied, the detailed mechanism of the forced unfolding has not been previously discussed. The existence of a stable 3-helix intermediate may offer yet another level in the mechanoregulation process and in the cell’s response to mechanical stimuli. The existence of the unfolding intermediate also adds additional complexity to the assessment of the impact of mutations in the case of diseases. α-catenin truncating mutations have been detected in patients with hereditary gastric cancer[46] possibly increasing the disease susceptibility. Furthermore, α-catenin mutations have been directly associated with macular dystrophy[47]. The understanding of molecular mechanisms would shed light on the disease development and guide new treatment solutions. We may also speculate that the existence of a 3-helix intermediate, whether undergoing full unfolding or not, may provide an additional structural support. It is also possible that the 3-helix state functions as a molecular bumper reducing the impact of functional mutations present in the mechanosensitive protein. In other words, with additional level of mechanoregulation, the mutation effect on the cells behavior may be defused with only moderate effect on the cells fitness. Such a theory may be of importance in the case of talin which has been presented as a vital protein in cell and tissue biology. Yet, despite its important roles, only one mutation has been recognized as disease causing, in the talin-2 isoform located outside of the mechanosensitive region[48].

We show that α-helical proteins unfold via stable 3-helix intermediate states, representing biologically active states. smAFM and disulphide clamp mutations were used to confirm the models obtained with SMD. Our results suggest that talin is a central scaffolding hub in focal adhesions with multiple discrete unfolding states, acting as a sophisticated mechanosensor and an important regulatory switch. We further propose that the mechanical stability of α-helical domains as well as the mechanical stability of their unfolding intermediates should be considered when studying mechanoregulation models of α-helical proteins.

## Materials and methods

### SMD simulations

The following structures from RCSB Protein Data Bank were used as the protein models for the individual talin rod subdomains: R3 (id 2L7A residues 796 to 909), R9 (id 2KBB) and R11 (id 3dYJ residues 1975 to 2140). Talin R9 tandems were constructed using PyMOL, by creating a peptide bond between the last residue of the first R9 monomer and the first residue of the second R9 monomer. α-catenin including M_I_ and M_II_ domains (id 4IGG residues 275 to 506) was used in our simulations. The point mutations introducing cysteine residues into the talin rod subdomains in order to form the disulphide bonds (clamps) preventing the unfolding of 3-helix state, were designed and mutated using PyMOL.

MD and SMD simulations were performed using Gromacs ver 2016.1[49,50] at the Sisu supercomputer, CSC, Finland. The CHARMM27 force field[51] and explicit TIP3P water model[52] in 0.15 M KCl solution were used and the total charge of the system was adjusted by K^+^ and Cl^-^ ions. The energy minimization of the system was performed in 10 000 steps using the steepest descent algorithm. The system was equilibrated in three phases using harmonic position restraints on all heavy atoms of the protein. The first phase of the equilibration was performed with NVT ensemble for 100 ps using the Berendsen weak coupling algorithm[53] to control the temperature of the system at 100 K. Integration time step of 2 fs was used in all the simulations. Following the NVT, the system was linearly heated from 100 to 310 K over 1 ns using an NPT ensemble at 1 atm of pressure. During this process, the Berendsen algorithm was used to control both temperature and pressure. For the final phase of equilibration and for all subsequent simulations, an NPT ensemble was maintained at 310 K using the V-rescale algorithm[54], and 1 atm as implemented in Gromacs 2016.1. The temperature coupling was applied separately for the protein and the solution parts. Each system was equilibrated up to 30 ns, with subsequent monitoring of the root mean square deviations (RMSD) of Cα atoms, considering the first approx. 5 ns as relaxation step. Hence, snapshots at 5 ns were used as starting structures for SMD simulations. Pulling vector was set between Cα of the first and the last residue of the appropriate domain. The movement of Cα of N-terminal residue was restrained with harmonic potential, while Cα of C-terminal residue was subjected to the constant velocity or constant force pulling. The pressure control was turned off for the pulling dimension (z-axes) in all SMD simulations as described in our previous work[28]. The constant velocity pulling SMD simulations were performed at 2 nm / ns with the spring constant set to 1000 kJ/mol nm^2^. In the constant force pulling SMD simulations, different force regimes were applied, 160 pN, 170 pN, 180 pN and 200 pN for R3 and 200 pN, 220 pN, 230 pN, 250 pN and 300 pN for R9. The system size in SMD was 227 thousand atoms for R3, about 800 thousand atoms for R9, R11 and α-catenin M_I_-M_II_, and about 1.2 million atoms for R9 tandems. Detailed composition of the systems used SMD simulations shown in S2 Table.

### Generation of polyprotein constructs

The constructs and experimental procedure for the smAFM were similar to those described before[28]. The talin fragment polyprotein constructs, including flanking I27, were synthesized and cloned in to pFN18a. The polyproteins were expressed in *E*. *coli* BL21-CodonPlus (DE3)-RILP competent cells, using the T7 promoter present in the plasmid. Protein expression was induced with IPTG when the culture reached an OD600 nm of 0.6. Cells were lysed by applying 0.2 mg/ml lysozyme for 30 minutes at 25°C, followed by sonication with an Sonifier cell disruptor model SLPe (Branson Ultrasonics Corporation, USA) and clarification of the lysate using centrifugation. The clarified lysate was subjected to Ni-NTA affinity chromatography beads in a batch process. The proteins eluted with imidazole were analyzed for purity with SDS-PAGE and used at a final concentration between 1–10 ug/mL.

### Preparation of ligand-functionalized surfaces

Glass coverslips were functionalized with the chloroalkane ligand to HaloTag as previously described[28]. The glass coverslips were first cleaned using Helmanex III (1% in water), acetone, and ethanol washes. The surfaces were then prepped with O_2_ plasma cleaning for 15 min. Surfaces were then silanized using (3-aminopropyl)trimethoxysilane, diluted to 1% in ethanol. Surfaces were then washed with ethanol and then dried with N_2_. These amine-functionalized surfaces were then incubated with 10 mM succinimidyl-[(N-maleimidopropionamido)tetracosaethylene glycol] ester (SMPEG24 –Thermo) diluted in 100 mM borax buffer (pH 8.5) for 1 h. The final step involved incubating the surfaces overnight with 10 mMHaloTag Thiol O4 ligand in the same buffer. The surfaces were quenched with 50 mM 2-mercaptoethanol in water.

### AFM experiments and analysis

We used a commercial AFS-1 from Luigs & Neumann, GmbH, based on a device developed at the Fernandez Lab, Columbia University[55]. The cantilevers used were gold-coated OBL-10 levers from Bruker. The spring constants varied between 4 and 10 pN/nm as measured by equipartition theorem with the appropriate adjustments for cantilever geometry[56,57].Around 20 μL of protein solution was incubated on functionalized coverslips for 30 min prior to the experiments to allow for HaloTag binding. The cantilever was pressed into the surface with a force of ∼300 pN to bind the cantilever to the polyprotein. Force extension experiments were conducted at 400 nm/s retraction rate. Data analysis was carried out using Igor Pro (Wavemetrics). Unfolding peaks were identified by adjustable smoothing with a moving box average and then by searching for local maxima. The force of the peaks along with their unadjusted distance from the HaloTag benchmark was measured.

## Supporting information

S1 Fig Design of disulphide clamps in R9. L1698C and A1748C cysteine mutations prevent unfolding of the 3-helix core from N-terminus (N clamp), while A1720C and A1779C mutations protect the 3-helix core from C-terminus (C clamp).
(TIF)

S2 Fig Statistical analysis of constant velocity SMD. Five parallel simulations were performed for (a) R9, (b) R11, (c) R3 and (d) α-catenin. Unfolding force profiles show that proposed mechanism of unfolding is reproducible.
(TIF)

S3 Fig Constant force SMD simulations in 100 ns time range. (a) R3 at constant force of 160 pN completely unfolded during ~ 72 ns, and (b) R9 unfolded to 3-helix state at 200 pN during ~ 86 ns.
(TIF)

S4 Fig Comparison between the folded and 3-helix intermediate state. Packing of hydrophobic residues in R3, R9 and R11 in (a, b & c) folded bundle (upper panel) and in (d, e & f) the 3-helix state (lower panel). The 3-helix structure snapshots were captured from constant velocity SMD simulations at 7 ns (R3) and 14 ns (R9 & R11). Side chains of hydrophobic residues are shown as orange sticks.
(TIF)

S5 Fig Analysis of R3 domain unfolding and corresponding end-to-end distance. The end-to-end distance of the folded bundle was measured in PyMol 1.7.x. The end-to-end distance of the collapsed R3 domain is hypothetical. It is based on the observation of the steered molecular dynamics under mechanical load. The length of intact helices was measured in PyMol 1.7.x. The final length of the unfolded state summed all measures of folded helices and contour lengths of the interconnecting linkers. The end-to-end distance of the theoretical unfolded R3 corresponds to the calculated contour length. 4 Å average length per residue was used in the theoretical length calculation (Ainavarapu et al. 2007).
(TIF)

S6 Fig Deletion of helix from talin rod subdomain R3 leads to enhanced accumulation to adhesion sites. Various mCherry-tagged talin forms were overexpressed in Talin-1 ^-/-^ mouse embryonal fibroblast cells [58]. Paxillin was used as a marker for the cellular adhesions. Wild type talin-1 and truncated (ΔR4-12) talin were found to co-localize with paxillin to similar extent. In contrast, talin ΔR4-12 4S containing destabilizing mutations in the R3 subdomain [23] and as well as talin ΔR4-12 ΔR3H4 having deletion of the last helix of R3 subdomain showed enhanced accumulation into paxillin-rich adhesion structures.
(TIF)

S1 Table Unfolding peak force in constant velocity SMD. (DOCX)

S2 Table Composition of the systems used in SMD simulations. (DOCX)
