# Supplementary figures and images for: Mechanical unfolding reveals stable 3-helix intermediates in talin and α-catenin
Source: PLoS Comput Biol. 2018 Apr 26;14(4):e1006126. doi: 10.1371/journal.pcbi.1006126 (PMC5940241; doi:10.1371/journal.pcbi.1006126)

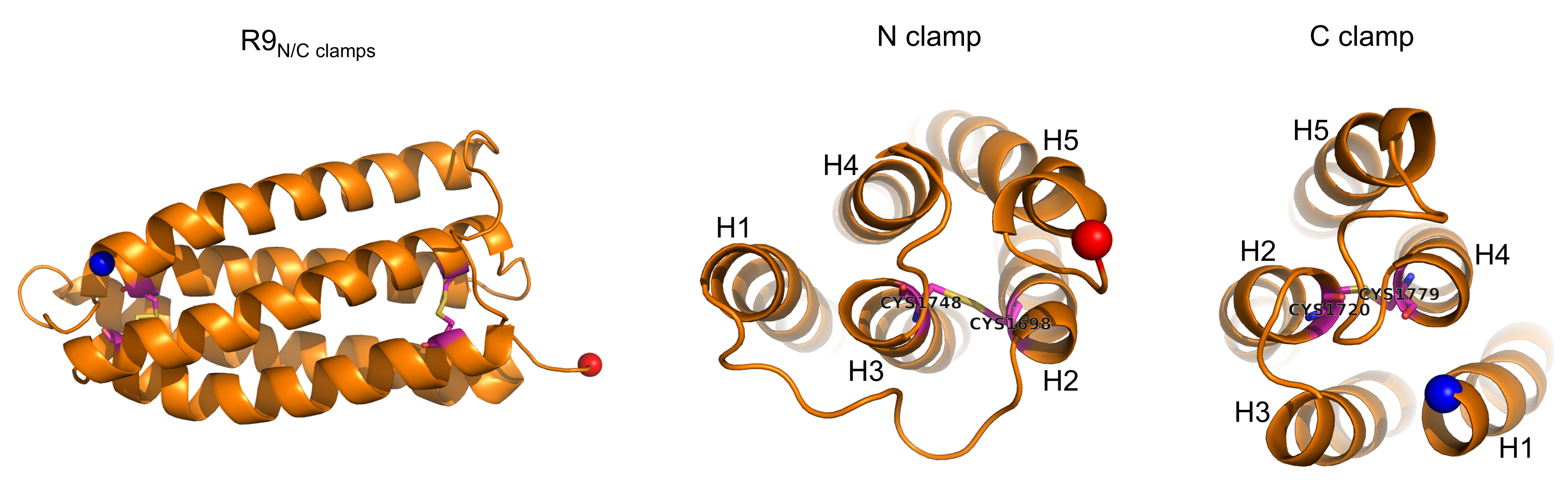

Supplement: S1 Fig — Design of disulphide clamps in R9. L1698C and A1748C cysteine mutations prevent unfolding of the 3-helix core from N-terminus (N clamp), while A1720C and A1779C mutations protect the 3-helix core from C-terminus (C clamp). (TIF) [file pcbi.1006126.s001.tif]

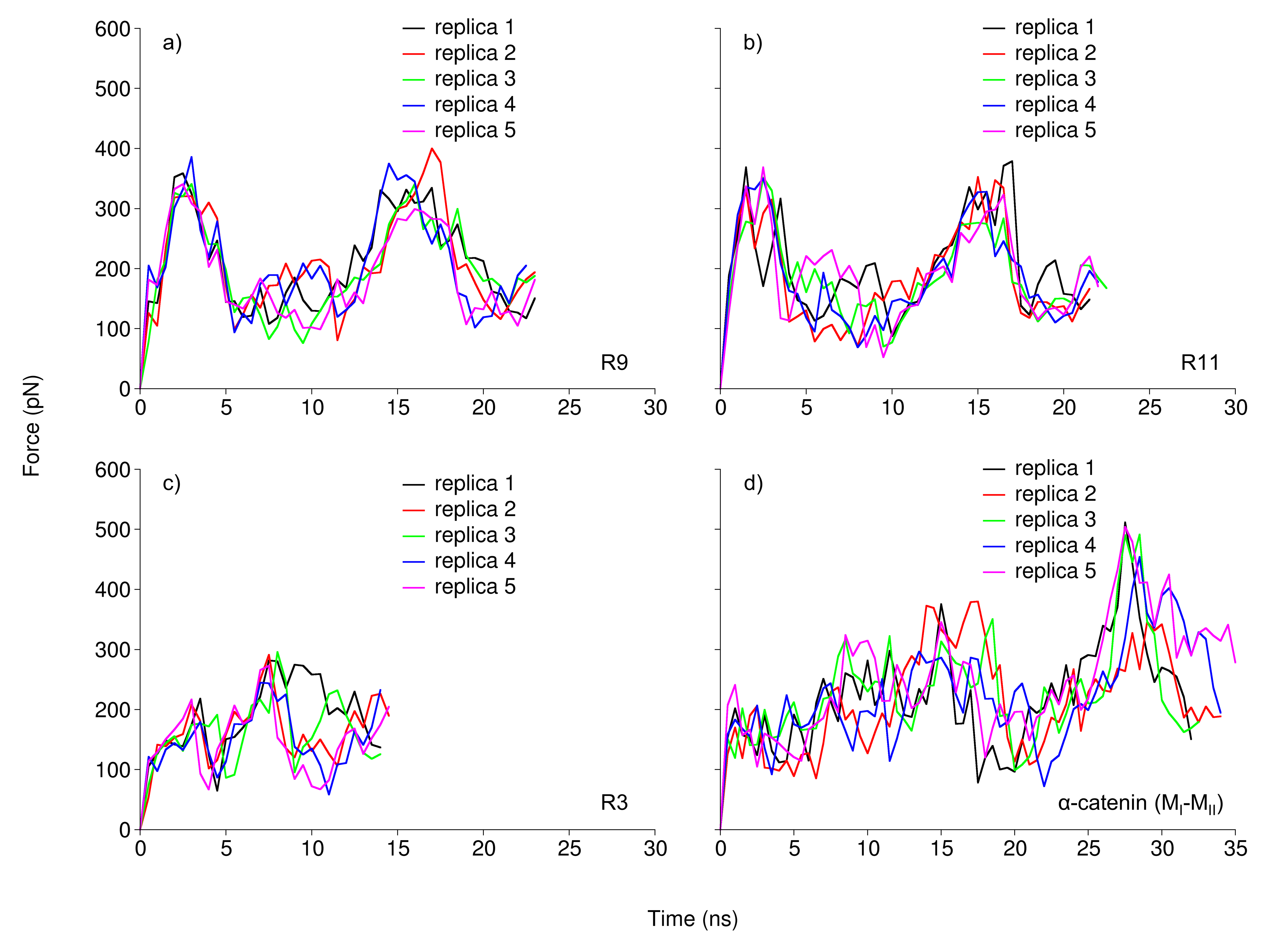

Supplement: S2 Fig — Five parallel simulations were performed for (a) R9, (b) R11, (c) R3 and (d) α-catenin. Unfolding force profiles show that proposed mechanism of unfolding is reproducible. (TIF) [file pcbi.1006126.s002.tif]

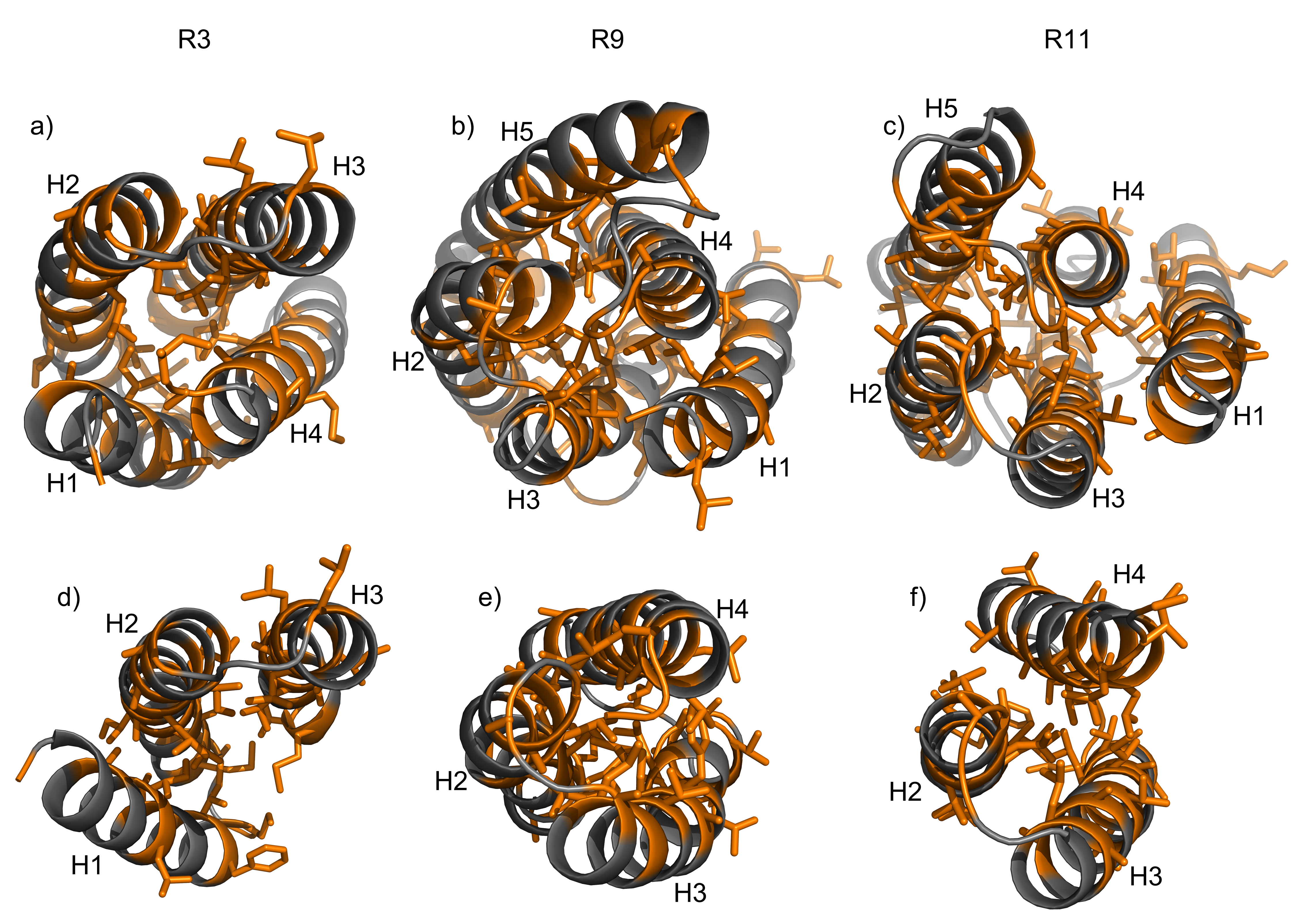

Supplement: S4 Fig — Packing of hydrophobic residues in R3, R9 and R11 in (a, b & c) folded bundle (upper panel) and in (d, e & f) the 3-helix state (lower panel). The 3-helix structure snapshots were captured from constant velocity SMD simulations at 7 ns (R3) and 14 ns (R9 & R11). Side chains of hydrophobic residues are shown as orange sticks. (TIF) [file pcbi.1006126.s004.tif]

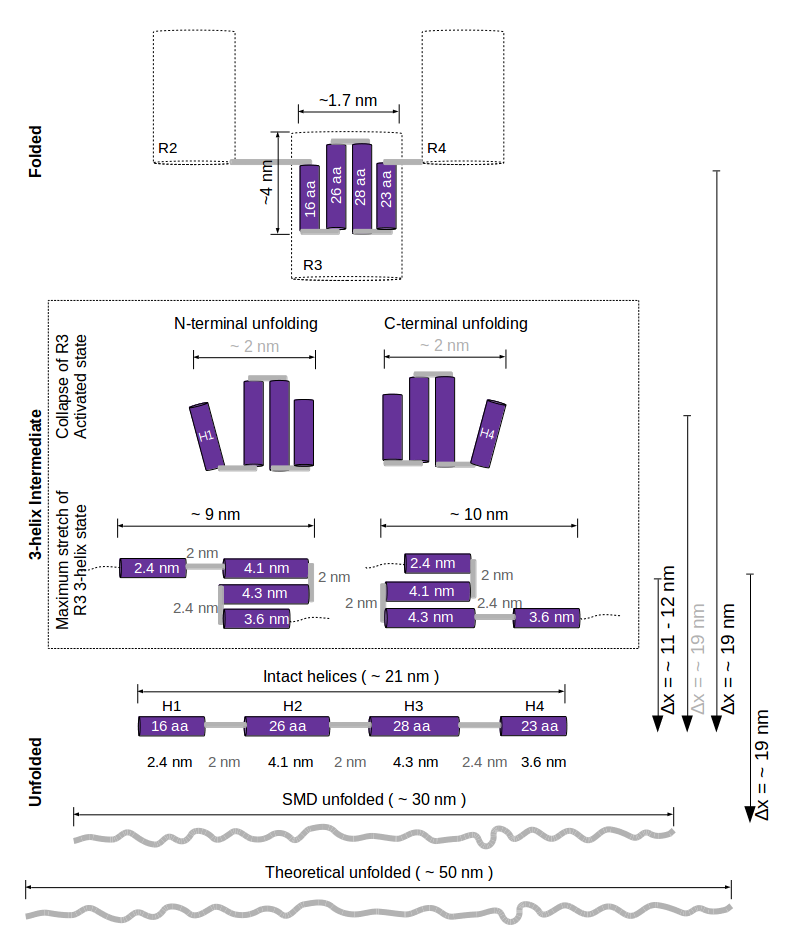

Supplement: S5 Fig — The end-to-end distance of the folded bundle was measured in PyMol 1.7.x. The end-to-end distance of the collapsed R3 domain is hypothetical. It is based on the observation of the steered molecular dynamics under mechanical load. The length of intact helices was measured in PyMol 1.7.x. The final length of the unfolded state summed all measures of folded helices and contour lengths of the interconnecting linkers. The end-to-end distance of the theoretical unfolded R3 corresponds to the calculated contour length. 4 Å average length per residue was used in the theoretical length calculation (Ainavarapu et al. 2007). (TIF) [file pcbi.1006126.s005.tif]

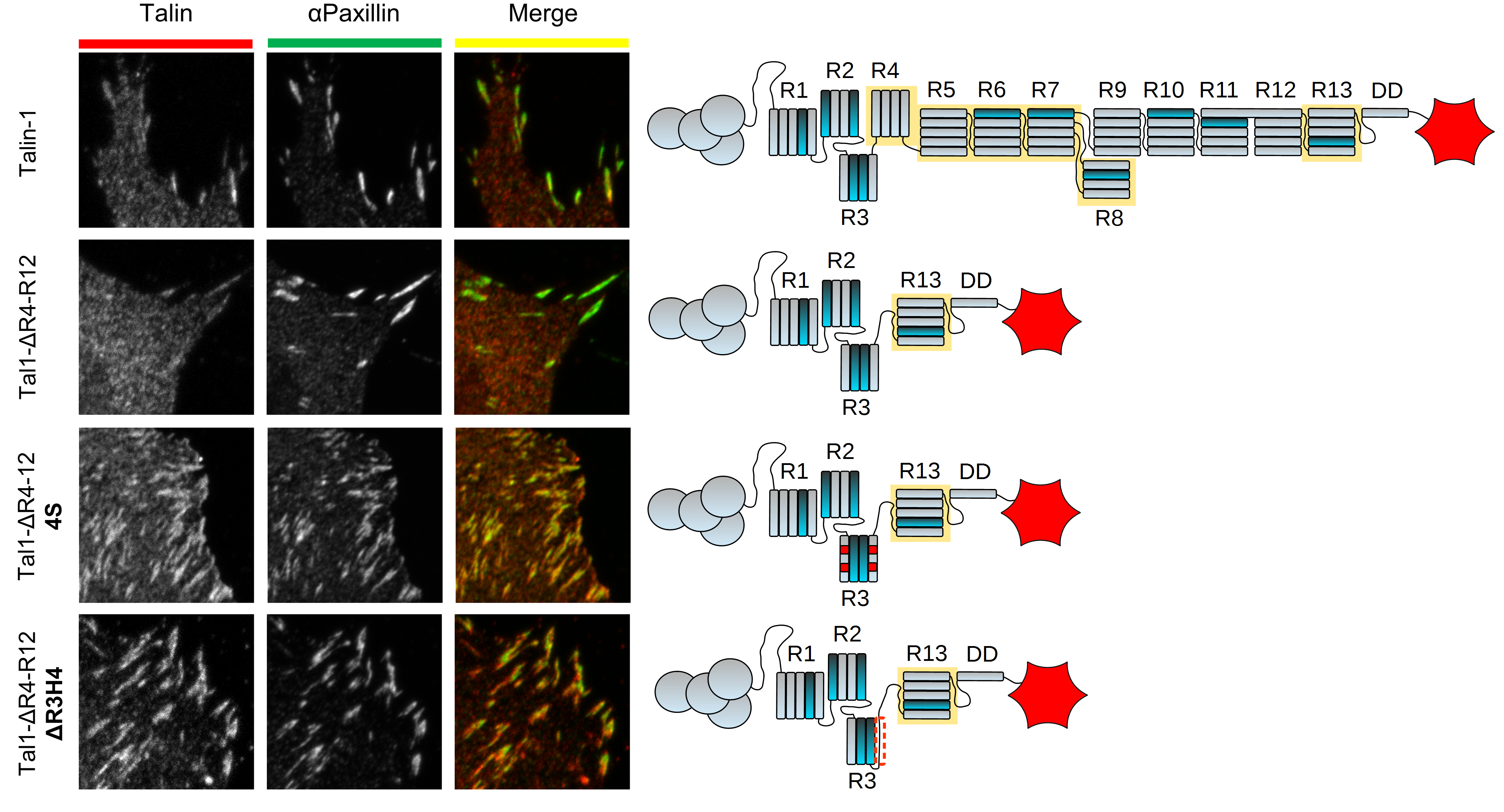

Supplement: S6 Fig — Various mCherry-tagged talin forms were overexpressed in Talin-1 -/- mouse embryonal fibroblast cells [58]. Paxillin was used as a marker for the cellular adhesions. Wild type talin-1 and truncated (ΔR4-12) talin were found to co-localize with paxillin to similar extent. In contrast, talin ΔR4-12 4S containing destabilizing mutations in the R3 subdomain [23] and as well as talin ΔR4-12 ΔR3H4 having deletion of the last helix of R3 subdomain showed enhanced accumulation into paxillin-rich adhesion structures. (TIF) [file pcbi.1006126.s006.tif]
