# Supplementary material for: Mechanical unfolding reveals stable 3-helix intermediates in talin and α-catenin
Source: PLoS Comput Biol. 2018 Apr 26;14(4):e1006126. doi: 10.1371/journal.pcbi.1006126 (PMC5940241; doi:10.1371/journal.pcbi.1006126)
Supplement: S1 Table — (DOCX) [file pcbi.1006126.s007.docx]

S1 Table. Unfolding peak force in constant velocity SMD

| Protein construct | Conformational state | Unfolding peak force (pN) | | | | | Average ± standard deviation |
| --- | --- | --- | --- | --- | --- | --- | --- |
|  |  | Replica 1 | Replica 2 | Replica 3 | Replica 4 | Replica 5 |  |
| R3 | 3h→0h | 281 | 291 | 295 | 244 | 272 | 276 ± 20 |
| R9 | 5h→3h | 358 | 320 | 340 | 385 | 340 | 348 ± 24 |
| R9 | 3h→0h | 331 | 400 | 340 | 374 | 299 | 349 ± 39 |
| R11 | 5h→3h | 368 | 329 | 351 | 349 | 368 | 353 ± 16 |
| R11 | 3h→0h | 378 | 352 | 283 | 327 | 322 | 332 ± 35 |
| α-catenin | 3h→0h (M_I_) | 511 | 345 | 491 | 454 | 504 | 461 ± 68 |
| α-catenin | 3h→0h (M_II_) | 375 | 379 | 350 | 296 | 345 | 349 ± 33 |
