# Supplementary material for: Mechanical unfolding reveals stable 3-helix intermediates in talin and α-catenin
Source: PLoS Comput Biol. 2018 Apr 26;14(4):e1006126. doi: 10.1371/journal.pcbi.1006126 (PMC5940241; doi:10.1371/journal.pcbi.1006126)
Supplement: S2 Table — (DOCX) [file pcbi.1006126.s008.docx]

S2 Table. Composition of the systems used in SMD simulations

| Protein construct | Residues | Number of protein residues (atoms) | Number of water molecules | Number of ions | | Total number of atoms |
| --- | --- | --- | --- | --- | --- | --- |
|  |  |  |  | K^+^ | Cl^–^ |  |
| R3 | Gly796-Ile909 | 114 (1672) | 75 236 | 214 | 208 | 227 802 |
| R9 | Gly1657-Gly1825 | 169 (2469) | 281 331 | 792 | 780 | 848 034 |
| R11 | Thr1975-Thr2140 | 166 (2429) | 272 147 | 758 | 754 | 820 382 |
| α-catenin (M_I_-M_II_) | Gly275-Thr506 | 232 (3655) | 237 484 | 665 | 659 | 717 431 |
